# A Case of Difficult Labor

**Published:** 1883-02-20

**Authors:** A. W. Reese


### A CASE OF DIFFICULT LABOR.

BY A. W. REESE, M. D.

On Saturday, October 16, 1871, I was hastily summoned, at ten o'clock p. m., to the bedside of Mrs. L., of this place. On my arrival I found present in the sick woman's room Dr. H. the attending physician, Dr. W. C., called in consultation first, and Dr. S. P., added to the council previous to my arrival at the house. I discovered it to be an unusual case of labor Dr. H., informed me that he had been called to the patient the preceding night (15th); that she had been in labor since two o'clock that morning; that she had just passed through two severe and alarming convulsions, and finally, that the medical gentlemen present all differed in regard to the presentation. Dr. H. gave it as his opinion that it was a presentation of the breech; Dr. W. C. thought it was the abdomen, and Dr. S. P. was sure that it was the shoulder of the fetus that presented itself to the touch.

Such then, in brief, was the history of the case, together with the differing views of the respectable medical men with whom I had been called to consult. The patient was profoundly under the influence of chloroform when I entered the room, Dr. H., the attending physician, administering the drug.

On concluding a statement of the above brief outlines of the case, Dr. H. asked me to make an examination and give my views, which I at once proceeded to do. The touch revealed a strange, unusually-shaped mass blocking up the entire pelvic cavity. In the course of a somewhat extensive obstetric practice, I had hitherto met nothing like it. And yet, in spite of its seemingly anomalous character, I could not resist the conviction that the vertex was the presenting part. In fact I felt sure that it was the head that came in contact under the touch.

The shape of this cumbrous mass was, I admit, altogether unlike any other fetal head I had ever met before, and I must confess that this fact was rather against than in favor of my diagnosis in this knotty case. But on the other hand I was sure that I could feel the short, silky hair that usually covers the fetal scalp.

Dr. S. P. could by no means agree with me in this diagnosis. He attributed the sensations derived by me from the touch, to abrasions of the cuticle upon the presenting part of the fetus, as there had been a good deal of manipulation before my arrival in the case. Neither my judgment nor experience could approve this view of the matter.

Whilst making my examination, I rapidly reviewed, in my own mind, the conflicting and diverse opinions of colleagues, and endeavored to determine their respective merits in a diagnostic point of view. First, then, I carefully scrutinized the position of Dr. H., the supposition of a breech presentation. And, truly, there seemed considerable grounds for his opinion. Here was a large mass, divided longitudinally, by a deep sulcus or groove, into two distinct, rather oblong hemispheres, which indeed bore a remarkable resemblance to the nates. But there were two features of the case which led me, unhesitatingly, to reject Dr. H.'s conclusion. In the first place, the hemispheres of the nates (if I may be allowed the expression) are soft, elastic, yielding, and pliable under the touch. In the present case these protuberances (whatever they might prove to be) were directly the reverse, being hard, dense, compact, inelastic, and, in short, felt to me just like bone covered by integument. Secondly, in passing my index finger from one end of this deep sulcus to the other (which, by the way, was no easy matter) I could discover neither the genital organs nor the anus; one or both of which must have been attainable in a breech presentation, except in a case of monstrosity, which latter is unusual and rare.

For these reasons I could not accept the view of the case under consideration as being a presentation of the breech. I next reviewed the opinion, expressed by Dr. W. C., that the abdomen was the portion of the fetus accessible to the touch. I endeavored to ascertain what features of the case could lead the Doctor's mind to this singular conclusion, for I had never met such a presentation, and was, moreover, skeptical as to its existence. The records of the profession sustain me in this opinion. Ramsbotham says, that in one hundred and fifty cases of transverse positions of the fetus, where he had been called to operate, he had met but one case of presentation of the abdomen. Chailly denies their existence altogether; though in the American edition of that author's work, Dr. Gunning Bedford, the editor, mentions a case of the kind which he saw in consultation. Cazeaux, Dubois, and Naegele recognize but two trunk presentations, one for the right and one for the left side. Madame Lachappelle denies the existence of such a presentation. This celebrated midwife declares that, in as many as forty thousand cases occurring at La Maternite, she had not met a single case of presentation of the abdomen.

In the case under consideration I could certainly expect to find either the soft, fluctuating, yielding parietes of the abdomen, the ensiform cartilage of the sternum, the symphysis pubis, or the insertion of the umbilical cord at the naval, if the belly, according to Dr. W. C., were the presenting part. But none of these portions of the fetus could be felt. I therefore excluded the abdomen from my diagnostic list. In confirmation of Dr. S. P's., theory, that it was the shoulder, I could feel neither the axilla, any portion of the arm, the clavicle, fetal ribs, neck, acromion process, nor any other evidence that would lead me to the conclusion that it was a presentation of the shoulder, right or left.

On retiring for consultation I gave my opinion, and the reasons influencing my mind in entertaining the views expressed. Each of my colleagues, however, seemed "fully persuaded in his own mind" of the correctness of his own diagnosis. Such being the case, not much concert of action could be expected.

Finally, after much talk, it was agreed to review the case, each of us to make another examination, and see what results could be obtained. We returned to the parturient chamber, and each one instituted a further examination, with the exception of Dr. H., the attending physician, who declined, stating that he was fully satisfied that it was the breech. Dr. W. C., the pioneer physician in the county, made a prolonged and rather tedious examination. He was succeeded by Dr. S. P., and lastly by the writer. We again retired for further consultation, and I must say that it was not a little amusing to see how positive each had become as to the correctness of his former opinion. No new light was thrown upon the subject.

Meantime it was becoming painfully evident that the vital forces of the patient were beginning to flag; the countenance was pale, the surface cool, the pulse feeble and growing quick and small, and the mind becoming despondent. These were serious symptoms, and showed that little time was to be lost in instituting some means for the woman's speedy relief.

The expulsive contractions of the uterus were powerful and continuous, but not a particle of advance was made by the presenting part of the fetus. As the result of further consultation resort was had to the forceps. Dr. S. P. volunteered his services in that direction, but after repeated and persistent efforts failed to deliver. After some time spent in these fruitless labors, the Doctor finally gave it up as a bad job, and asked me to try my hand. I took the handles of the forceps and withdrew the instrument from the patient's body. In reply to the Doctor's expressive look of inquiry, I said, "I am loth to use the forceps when there is room for doubt as to what part of the fetus they are to be applied."

I then made the third examination, as did also the two other consulting physicians, but without coming any nearer to an agreement than at first. I then made the suggestion that, regardless of the presentation, an effort should be made to reach the feet, and by turning the fetus deliver at once. This proposition met with general favor, for we had now reached a stage in the proceedings when anything looking toward relief was gladly accepted. I was requested to make the attempt. I did so, and after great difficulty succeeded in reaching the feet, but found it impossible to turn. The two remaining consulting physicians both made similar efforts but without success.

At this stage of the case a final consultation was held, in which it was determined to use the perforator at once upon the most accessible part of the fetus, regardless of what it might ultimately prove to be, and thus by materially reducing its bulk, effect the speedy delivery of the woman, whose condition was now beyond question one of extreme peril. The perforator was therefore immediately brought into requisition. This procedure was instantly followed by an immense gush o water, a gallon at least in quantity, making its escape in a literal torrent. The blunt hook being then inserted into the opening made by the perforator, the fetus was speedily brought through the vulva. An inspection revealed a very large child with an enormously enlarged head.

The incision made by the perforator was directly in the center of the median line between the os frontis and the occiput, through the sagittal suture, thus putting the question of the presentation beyond all dispute. The sulcus felt by us was caused by the terrific pressure brought to bear by the uterine contractions upôn the parietal bones of the fetal head.

On measurement, which was effected by stuffing the cranial cavity with raw cotton and the use of a tape line, the proportions of the fetal head were found to be enormous. The distance from the nasal bones to the occipital protuberance was twenty-two and one half inches; circumference, measuring just above the ears, twenty-nine inches; from apex of chin to anterior fontanelle, nineteen inches. Unfortunately we neglected to weigh the child, but it could not have fallen short of fourteen or fifteen pounds. With the exception of its head it was well shaped and healthy in appearance.

I saw this patient again on Sunday morning, the 17th, in consultation. Dr. W. C. and I called together. The symptoms were regarded as unfavorable. There was a pulse of one hundred and ten, inclining to be small and wiry, hypogastric tenderness, and pain and slight tympanitis. She was rational but restless and despondent. Dr. H. was still in charge of the case. I expressed my opinion to Dr. W. C., on our departure from the house, that she could not survive. The Doctor coincided in this view of the case. The prognosis proved correct, for she grew rapidly worse, and in a few days perished from metroperitoneal inflammation.

I am led to report this melancholy case, not for any purpose of self-glorification, not because I claim any special infallibility in diagnosis, or that I desire to appear "wise above that which is written," but because I think it an instructive case that may prove of some benefit to the profession, especially its junior members, and that there is sometimes "in a multitude of counsel" considerable confusion.

Accuracy in diagnosis is not always possible, even to the most experienced and skillful men. Mistakes will sometimes occur even with the best informed members of the healing art. I am satisfied that cases do arise where the wisest heads are sorely puzzled. Skill in diagnosis is the result of patient, laborious, and careful observation. Abernathy once said that "genius in a medical man consists in a patient observation of facts."

A man is a physician in the highest sense of the name, just in proportion to his knowledge of pathology and his skill in diagnosis, for "upon these two hang all the law and the *profits*." (Excuse the pun.)

The more we are impressed with this fact the more certainly shall we approximate that perfection in our noble profession which is the goal of our common ambition.—*Louisville Medical News.*

---

**Salicylic Acid in Psoriasis and Eczema.**—Dr. I. Rabitsch, of Cairo, speaks very highly of a ten per cent. solution of salicylic acid in forty per cent. alcohol for the treatment of psoriasis, eczema, and especially the different varieties ot tiniæ. He records a number of cases, and claims that it is an excellent parasiticide.

